# Prognostic Significance of Left Ventricular Global Work Efficiency in Obese Patients with Acute ST-Segment Elevation Myocardial Infarction—A Pilot Study

**DOI:** 10.3390/diagnostics15121512

**Published:** 2025-06-14

**Authors:** Alexandra-Cătălina Frișan, Marius Simonescu, Mihai-Andrei Lazăr, Simina Crișan, Aniko Mornoș, Raluca Șoșdean, Andreea-Roxana Morar, Daniel-Miron Brie, Constantin-Tudor Luca, Cristian Mornoș

**Affiliations:** 1 Cardiology Department, “Victor Babeș” University of Medicine and Pharmacy, 2 Eftimie Murgu Square, 300041 Timișoara, Romania; 2 Research Center of the Institute of Cardiovascular Diseases Timisoara, 13A Gheorghe Adam Street, 300310 Timișoara, Romania; 3 Institute of Cardiovascular Diseases Timisoara, 13A Gheorghe Adam Street, 300310 Timișoara, Romania

**Keywords:** myocardial infarction, obesity, myocardial work, global work efficiency, echocardiography, major adverse cardiac events, global longitudinal strain, speckle tracking

## Abstract

**Background/Objectives:** Obesity is increasingly common among patients with acute ST-segment elevation myocardial infarction (STEMI), potentially influencing both clinical evaluation and outcomes. Traditional echocardiographic metrics may be suboptimal for prognosis estimation in this population. Left ventricular myocardial work (LVMW) represents an emerging, load-adjusted marker of myocardial performance. This study aimed to assess the prognostic relevance of LVMW in obese STEMI patients. **Methods:** A total of 143 patients presenting with STEMI were prospectively enrolled and categorized based on their obesity status (body mass index ≥30 kg/m^2^). LVMW parameters were measured using echocardiography within 72 ± 24 h of hospital admission. The patients were monitored for major adverse cardiovascular events (MACE), defined as cardiovascular death, malignant ventricular arrhythmias, or unplanned hospitalizations due to heart failure or acute coronary syndrome. **Results:** During a median follow-up of 13 months (interquartile range: 6–28 months), MACE occurred in 30 patients (21%). Among obese individuals, left ventricular global work efficiency (LVGWE) emerged as the most robust predictor of adverse events, with an area under the receiver operating characteristic curve of 0.736 (95% confidence interval [CI]: 0.559–0.914; *p* = 0.009). A threshold value of 79% for LVGWE was identified as optimal for predicting MACE. Kaplan–Meier analysis revealed significantly lower event rates in obese patients with LVGWE ≥79% (log-rank *p* = 0.006). In univariate Cox regression analysis, LVGWE <79% was associated with a markedly elevated risk of MACE in obese patients (hazard ratio [HR] = 5.59; 95% CI: 1.33–23.50; *p* = 0.019), and remained a significant predictor in the overall cohort (HR = 2.73; 95% CI: 1.26–5.90; *p* = 0.010). **Conclusions:** LVGWE demonstrates strong prognostic utility in STEMI, particularly among obese patients. The incorporation of myocardial work indices into routine evaluation may enhance risk stratification and guide management in this high-risk subgroup.

## 1. Introduction

Obesity, defined as a body mass index (BMI) ≥30 kg/m^2^, has emerged as a global health epidemic with steadily rising prevalence and significant cardiovascular consequences [1]. Current projections suggest that nearly two-thirds of adults aged ≥25 years will be affected by overweight or obesity in the near future [2].

Obesity triggers complex metabolic derangements that adversely affect myocardial function. A key alteration is the shift toward predominant fatty acid oxidation, accompanied by impaired glucose utilization. Since fatty acid metabolism is less oxygen-efficient, the myocardium requires increased oxygen consumption to generate equivalent amounts of adenosine triphosphate, thereby reducing mechanical efficiency. This maladaptive shift results in greater myocardial oxygen demand without proportional enhancement in contractile performance. Moreover, excessive fatty acid oxidation promotes the accumulation of lipotoxic intermediates, such as ceramides and diacylglycerol, which contributes to myocardial insulin resistance, impaired calcium handling, and reduced contractility [3,4,5]. These metabolic abnormalities are often accompanied by structural cardiac changes frequently observed in obesity, including elevated filling pressures, left atrial enlargement, and left ventricular (LV) hypertrophy, further aggravating myocardial dysfunction [6].

Clinical data indicate that patients with acute myocardial infarction and BMI ≥30 kg/m^2^ are at increased risk for adverse outcomes, including heart failure-related rehospitalization and cardiovascular death [7]. However, the relationship between BMI and outcomes is nuanced and varies with the specific clinical endpoint. These observations highlight the limitations of BMI as a solitary prognostic marker and underscore the need for individualized risk stratification strategies in post-infarction care.

Although LV ejection fraction (LVEF) remains the most widely used echocardiographic parameter for assessing systolic function—even in individuals with obesity [8]—it may not fully capture the complexity of myocardial injury, particularly in acute settings, such as ST-segment elevation myocardial infarction (STEMI). Advanced echocardiographic modalities have improved the assessment of myocardial function [9]. Left ventricular myocardial work (LVMW), derived from non-invasive pressure–strain loop analysis, integrates deformation imaging with blood pressure data to provide a more physiologic assessment of myocardial performance [10,11,12,13].

Among myocardial work indices, left ventricular global work efficiency (LVGWE) has gained increasing attention as a comprehensive marker of cardiac performance [14]. LVGWE reflects the proportion of total myocardial work that contributes to effective ejection, discounting energy wasted during isovolumic contraction and relaxation [15]. By integrating afterload and myocardial deformation into a single, load-adjusted index, LVGWE offers a physiologically meaningful measure of LV function that may better capture early or subclinical dysfunction, particularly in pathophysiologic states, such as obesity, where standard metrics like LVEF may be preserved despite underlying inefficiency [16].

Given these considerations, we hypothesized that LVMW indices, particularly LVGWE, could provide incremental prognostic value in obese patients presenting with STEMI. The objective of this study was to evaluate the predictive utility of LVMW parameters for major adverse cardiac events (MACEs) in this high-risk population.

## 2. Materials and Methods

### 2.1. Study Population

This prospective observational study included 150 consecutive patients with acute STEMI admitted to our tertiary center over a 12-month period. All underwent primary percutaneous coronary intervention (PCI) within 12 h of symptom onset and received a comprehensive transthoracic echocardiographic (TTE) evaluation. The exclusion criteria were significant primary valvular disease, atrial fibrillation or significant R–R interval variability, presence of a pacemaker, life expectancy <1 year due to comorbid conditions, or suboptimal echocardiographic image quality. After exclusions, 143 patients were included in the final analysis.

The patients were stratified based on BMI at admission:

- Group 1: Non-obese (BMI < 30 kg/m^2^);
- Group 2: Obese (BMI ≥ 30 kg/m^2^).

All patients received evidence-based treatment per current STEMI guidelines [17] and were followed longitudinally in a structured outpatient cardiology program. The primary endpoint was the occurrence of MACE, defined as a composite of unplanned cardiovascular hospitalization (for heart failure or acute coronary syndrome), malignant ventricular arrhythmias, or cardiovascular death.

The study protocol was approved by the institutional ethics committee (approval No. 10163/5 December 2023) and the university ethics board (No. 07/31 January 2024). All participants provided written informed consent.

### 2.2. Demographic and Clinical Characteristics

Demographic and clinical data, including age, sex, heart rate and blood pressure on admission, BMI, and body surface area, were extracted from electronic medical records. Cardiovascular risk factors, such as smoking status, diabetes mellitus, hypertension, dyslipidemia, and family history of cardiovascular disease, were also recorded. The biochemical parameters assessed included fasting glucose, creatinine, hemoglobin, leukocyte and neutrophil counts, total cholesterol, low-density lipoprotein cholesterol, triglycerides, erythrocyte sedimentation rate, and peak levels of myocardial necrosis biomarkers (high-sensitivity troponin I and creatine kinase–MB).

### 2.3. Echocardiographic Assessment

All patients underwent a TTE within 72 ± 24 h of admission using a Vivid 9 ultrasound system (GE Vingmed Ultrasound, Milwaukee, Brookfield, WI, USA) equipped with an M5S probe. Images were acquired in the left lateral decubitus position at rest, and electrocardiographic gating was used. Blood pressure was measured immediately prior to the examination to estimate LV systolic pressure. All echocardiographic measurements followed the current recommendations of the European Association of Cardiovascular Imaging and the American Society of Echocardiography [18].

Tissue Doppler imaging was performed in pulsed-wave mode from the apical four-chamber view, using a high frame rate (typically 150–200 frames per second) to ensure optimal temporal resolution for myocardial velocity measurements. Peak early diastolic velocity (e’), late diastolic velocity (a’), and systolic velocity (s’) were recorded from the septal and lateral mitral annulus. Signals were acquired at end-expiratory apnea over five consecutive cardiac cycles and averaged. A sweep speed of 100 mm/s was used. The average of septal and lateral segmental values was used for analysis.

For the speckle-tracking echocardiography, standard apical four-, two-, and three-chamber views were used. Global longitudinal strain (GLS) was automatically calculated and adjusted manually when necessary to ensure accurate delineation of endocardial borders. Segments that could not be tracked were excluded. Any apical view with two or more non-trackable segments was omitted from the analysis. The remaining apical views were then averaged to calculate GLS.

### 2.4. Myocardial Work Analysis

LVMW indices were computed using the EchoPac software (version 206, GE Vingmed Ultrasound, Horten, Norway). The opening and closing times of the mitral and aortic valves were determined using continuous-wave Doppler recordings, visually verified in the apical three-chamber view, and manually adjusted when necessary. The software generated a pressure–strain loop using GLS and brachial cuff systolic blood pressure.

The following parameters were calculated:

- LV Global Work Index (LVGWI)—total myocardial work from mitral valve closure to opening;
- LV Global Constructive Work (LVGCW)—work contributing to LV ejection (systolic shortening and isovolumic relaxation lengthening);
- LV Global Wasted Work (LVGWW)—energy expenditure that does not contribute to ejection (systolic lengthening and isovolumic shortening);
- LV Global Work Efficiency (LVGWE)—calculated as follows:

LVGWE = LVGCW/(LVGCW + LVGWW) × 100%

Echocardiographic assessments were independently performed by two experienced cardiologists blinded to the clinical data.

### 2.5. Statistical Analysis

The normality of the data distribution was assessed using the Shapiro–Wilk test. Categorical variables are reported as frequencies and percentages. Continuous variables are expressed as the mean ± standard deviation for normally distributed data or as the median (interquartile range) for skewed distributions. Group comparisons were made using the chi-squared or Fisher’s exact test for categorical variables, and the independent t-test or Mann–Whitney U test for continuous variables, as appropriate.

Receiver operating characteristic (ROC) analysis and the Youden index were used to determine the optimal cut-off values of LVMW indices for predicting MACE. Kaplan–Meier survival analysis and the log-rank test were used to compare outcomes. Cox proportional hazards regression was performed to identify independent predictors of adverse events. Spearman correlation analysis was performed using BMI as a continuous variable to explore potential linear relationships with LVGWE.

Intraclass correlation coefficients were calculated to assess intra- and interobserver variability, based on a random sample of 21 patients. Intraobserver variability was assessed by repeating measurements at two time points, while interobserver agreement was determined by comparing results from two independent readers.

Statistical analysis was performed using SPSS (version 29.0.2.0, IBM Corp., Armonk, NY, USA). A two-tailed *p*-value < 0.05 was considered statistically significant.

## 3. Results

### 3.1. Characteristics of the Patient Population

A total of 143 patients (77.7% male; mean age of 59 ± 11 years) who met the inclusion criteria were enrolled in the study and underwent TTE evaluation within 72 ± 24 h of hospital admission. Thrombolytic therapy prior to angiocoronarography was administered in 39 patients (27.3%).

Coronary angiography revealed single-vessel disease in 63 patients, two-vessel disease in 47, and three-vessel disease in 33. Infarct localization showed anterior STEMI in 38.5% of cases, inferior in 51.7%, posterolateral in 4.2%, and lateral in 5.6%. All patients underwent successful PCI within 12 h of symptom onset.

Based on BMI classification [19], 38 patients (26.6%) had class I obesity (BMI 30.0–34.9 kg/m^2^), 9 patients (6.3%) had class II obesity (BMI 35.0–39.9 kg/m^2^), and 6 patients (4.2%) had class III obesity (BMI ≥ 40 kg/m^2^), corresponding to morbid obesity.

During a median follow-up of 13 months (interquartile range: 6–28 months), 30 patients (21%) experienced an adverse outcome, including 3 deaths (2.1%), 22 re-hospitalizations due to decompensated heart failure or acute coronary syndrome (15.4%), and 5 cases of malignant ventricular arrhythmia (3.5%). The incidence of MACE did not differ significantly between obese and non-obese patients (15.1% vs. 24.4%, *p* = 0.185).

Baseline clinical characteristics and laboratory parameters are summarized in Table 1. In the overall cohort of 143 patients, the average systolic and diastolic blood pressures at admission were 146 ± 23 mmHg and 89 ± 15 mmHg, respectively, with a mean heart rate of 82 ± 16 bpm. The average BMI was 28.8 kg/m^2^. Elevated fasting glucose levels were observed, with a mean value of 134 mg/dL. Regarding cardiovascular risk factors, there was a high prevalence of dyslipidemia (95.8%), hypertension (75.5%), and smoking (68.5%). Most patients presented in Killip class I at admission (83.9%).

Compared with non-obese individuals, patients with a BMI ≥ 30 kg/m^2^ were significantly younger and exhibited higher systolic and diastolic blood pressure, as well as elevated blood glucose and triglyceride levels. Additionally, obesity was associated with a higher prevalence of hypertension and diabetes mellitus. No significant differences were observed between the two groups regarding sex distribution, heart rate, serum creatinine, estimated glomerular filtration rate, low-density lipoprotein cholesterol, erythrocyte sedimentation rate, or peak concentrations of cardiac necrosis biomarkers.

During hospitalization, patients with STEMI and obesity were more frequently prescribed beta-blockers and renin–angiotensin–aldosterone system inhibitors. No significant differences were observed between obese and non-obese patients regarding the prescription of other evidence-based cardiovascular medications (Table 2). Consistent with our institution’s treatment protocol and current clinical guidelines [17], neprilysin inhibitors (ARNIs) were not utilized in the management of patients during hospitalization at the time of study enrollment.

### 3.2. Echocardiographic Results

Echocardiographic image quality was adequate for quantitative analysis in all patients ultimately included in the study. Although mild image degradation was more frequently observed in obese individuals, it did not hinder the acquisition of essential measurements in any case. Notably, obesity remains a recognized challenge for transthoracic echocardiography; in this context, five patients presenting with suboptimal image quality were excluded from the final analysis. Compared to non-obese STEMI patients, those with obesity exhibited significantly higher LV end-diastolic volume, end-systolic volume, interventricular septal thickness, posterior LV wall thickness, and late diastolic transmitral flow velocity (A wave). However, there were no significant differences between the two groups in terms of LVEF or GLS (Table 3).

Regarding the LVMW parameters, obese patients demonstrated a significantly reduced LVGWE and increased LVGWW compared to their non-obese counterparts (Table 4).

### 3.3. Receiver Operating Characteristics (ROC) Analysis

The ROC analysis was conducted to evaluate the predictive performance of various echocardiographic parameters for identifying MACEs in obese and non-obese STEMI patients. Among the parameters evaluated, LVGWE demonstrated the highest predictive value for adverse outcomes in obese STEMI patients (Table 5), with an area under the curve (AUC) of 0.736 (95% confidence interval [CI]: 0.559–0.914; *p* = 0.009). The optimal cutoff value for LVGWE, determined by the Youden index, was 79%, with a sensitivity of 62% and a specificity of 82%. In contrast, none of the evaluated parameters achieved statistical significance in predicting MACEs among non-obese STEMI patients (Table 6).

**Figure 1:**
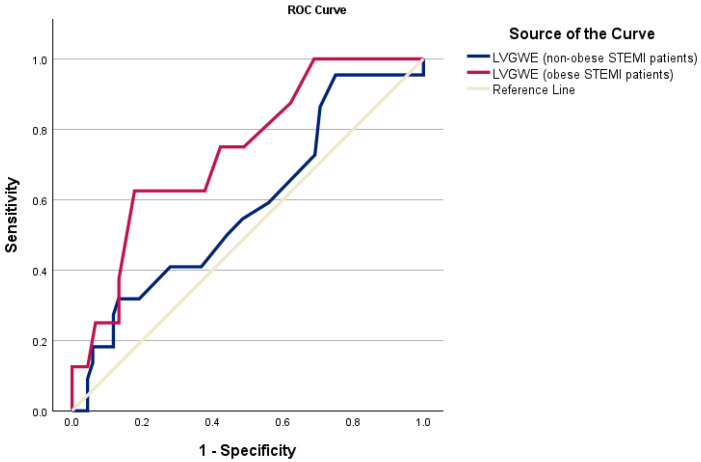
ROC curves of LVGWE for detecting major adverse cardiac events in non-obese (blue line) and obese STEMI patients (red line). ROC, receiver operating characteristic; LVGWE, left ventricular global work efficiency; STEMI, ST-elevation myocardial infarction.

Figure 1 illustrates the ROC curves of LVGWE for detecting MACEs in obese and non-obese STEMI patients.

### 3.4. Kaplan–Meier Survival Analysis

**Figure 2:**
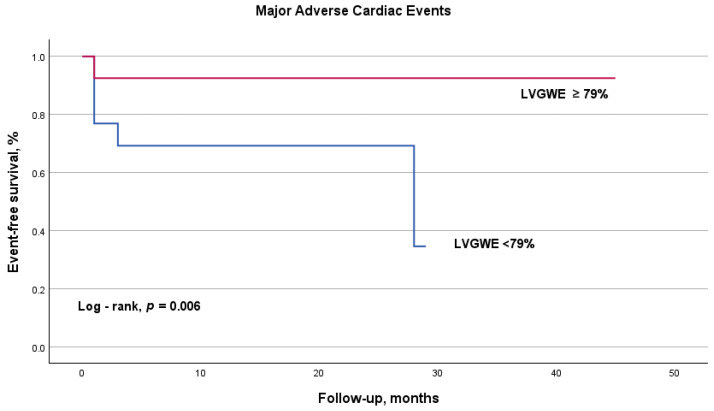
Kaplan–Meier curve for major adverse cardiac events in obese STEMI patients according to the cutoff value of left ventricular global work efficiency. The curve illustrates improved survival rates for patients with LVGWE ≥ 79% (red line) compared to those with LVGWE < 79% (blue line) (log-rank, *p* = 0.006). LVGWE, left ventricular global work efficiency.

Kaplan–Meier survival analysis showed that obese STEMI patients with a LVGWE ≥ 79% exhibited a significantly better prognosis compared to those with lower LVGWE values, as demonstrated by the log-rank test (*p* = 0.006). These findings suggest that LVGWE serves as a meaningful prognostic marker, effectively distinguishing between patients with favorable and poorer outcomes within the obese STEMI cohort (Figure 2).

### 3.5. Cox Regression Analysis

In obese STEMI patients, univariate Cox proportional hazards regression revealed a significant association between LVGWE—using a cutoff value of 79%—and clinical outcomes (hazard ratio [HR]: 5.59; 95% CI: 1.33–23.50; *p* = 0.019), indicating an increased risk of adverse events for those with LVGWE < 79%. Specifically, patients with LVGWE < 79% experienced a 5.59-fold higher risk of adverse outcomes compared to those with LVGWE ≥ 79% (Table 7).

To assess the broader prognostic value of the LVGWE cutoff, we extended the analysis to the entire study population (Table 8). In both univariate and multivariate models, LVGWE < 79% remained a significant predictor of adverse clinical outcomes. This association persisted even after adjustment for key confounders, including obesity status and infarct size, as reflected by peak creatine kinase-MB levels (adjusted HR: 2.73; 95% CI: 1.26–5.90; *p* = 0.010). These findings suggest that the prognostic utility of LVGWE extends beyond the obese population, supporting its potential value as a valuable risk stratification tool in the broader STEMI population.

### 3.6. Correlation Analysis

To explore whether a linear relationship existed between BMI and LVGWE, we performed a Spearman correlation analysis using both BMI and LVGWE as continuous variables. The analysis demonstrated a weak inverse correlation (r = −0.147), which did not reach statistical significance (*p* = 0.079).

### 3.7. Reliability of MW Parameters

All MW parameters demonstrated excellent intraobserver agreement, with intraclass correlation coefficients as follows: 0.987 (95% CI: 0.967–0.995) for LVGWI, 0.986 (95% CI: 0.965–0.994) for LVGCW, 0.974 (95% CI: 0.935–0.989) for LVGWW, and 0.975 (95% CI: 0.939–0.990) for LVGWE. Interobserver variability was also satisfactory for all MW indices, with the following values: 0.968 (95% CI: 0.923–0.987) for LVGWI, 0.962 (95% CI: 0.910–0.984) for LVGCW, 0.963 (95% CI: 0.913–0.985) for LVGWW, and 0.962 (95% CI: 0.909–0.984) for LVGWE.

## 4. Discussion

The present study investigated the prognostic significance of myocardial work indices in patients with acute STEMI, with a particular focus on individuals with obesity. Our data demonstrates that LVGWE serves as a robust predictor of adverse outcomes in STEMI patients, particularly among those with BMI ≥ 30 kg/m^2^.

Notably, the threshold of LVGWE < 79% reliably identified a subgroup at increased risk for MACEs, making it a promising prognostic marker. This cut-off remained prognostically relevant even when applied to the broader cohort, independent of obesity and infarct size, thereby underscoring its potential value in comprehensive STEMI risk stratification.

### 4.1. Prognostic Value of LVGWE in STEMI

Numerous studies have examined echocardiographic parameters in relation to STEMI prognosis. Traditionally, LVEF has been the cornerstone for assessing post-infarction systolic function and outcome prediction [20,21,22]. However, technological advances in echocardiography—particularly speckle-tracking and tissue Doppler imaging—have enabled a more sophisticated evaluation of myocardial performance. Among emerging modalities, GLS and myocardial work indices have shown superior prognostic performance [23,24,25,26].

Our findings reinforce this paradigm shift, demonstrating that LVGWE surpasses LVEF in predicting adverse outcomes in STEMI patients. Neither LVEF nor other conventional metrics reached statistical significance in univariate or ROC analyses, whereas LVGWE emerged as a robust prognostic marker. This superiority is biologically plausible: LVGWE reflects the proportion of total myocardial work that contributes to effective ejection, correlating more closely with myocardial oxygen consumption and energy efficiency than LVEF [27,28,29].

Comparable results have been reported in prior investigations. Lustosa et al. showed that an LVGWE < 86% independently predicted all-cause mortality in STEMI, with added prognostic value over LVEF and GLS [30]. Similarly, Coisne et al. identified an LVGWE threshold of 91% as predictive of adverse events one month after acute myocardial infarction, enhancing early post-acute myocardial infarction risk stratification [31].

Our study adds to this growing body of evidence by demonstrating that a lower LVGWE (<79%) is especially informative in obese STEMI patients. The heterogeneity in threshold values reported across studies likely reflects differences in population characteristics, infarct severity, timing of echocardiographic assessment, and methodological variability, underscoring the need for further research to refine clinically applicable cut-offs.

### 4.2. Impact of Obesity on STEMI Outcomes

We also observed that obese patients with STEMI had significantly lower LVGWE values than their non-obese counterparts, aligning with established pathophysiological mechanisms linking obesity to myocardial dysfunction [32,33,34,35].

Obesity contributes to an adverse myocardial metabolic profile, characterized by increased fatty acid oxidation, impaired glucose metabolism, elevated myocardial oxygen consumption, and structural remodeling [4], all of which compromise contractile efficiency and increase vulnerability to ischemic injury.

Consistent with previous studies, our obese subgroup exhibited a higher prevalence of cardiometabolic comorbidities, including hypertension, diabetes mellitus, and dyslipidemia, as well as elevated systolic and diastolic blood pressures and hyperglycemia—all known contributors to adverse outcomes in the context of STEMI [36,37].

Obesity is also associated with increased risks of heart failure, arrhythmias, and cardiovascular mortality in patients with acute coronary syndromes [38,39].

Our findings support the hypothesis that LVGWE captures obesity-related myocardial dysfunction beyond conventional risk factors. Its ability to detect subclinical inefficiencies in myocardial performance may render it particularly useful for identifying obese STEMI patients at high risk of complications, potentially guiding targeted therapeutic strategies and resource allocation.

Although our primary analysis was based on BMI categories, an exploratory assessment using BMI as a continuous variable did not reveal a statistically significant association with LVGWE. These findings highlight the complexity of the relationship between adiposity and myocardial work efficiency and suggest that additional parameters beyond BMI may be needed to capture this relationship more accurately.

### 4.3. Limitations and Future Directions

Despite its strengths, this study has limitations that must be acknowledged. The relatively small sample size, particularly within the obese subgroup, limited the statistical power and precluded robust multivariate analyses in that group. Additionally, the short-to-intermediate follow-up duration (median 13 months) may not fully capture long-term prognostic implications of myocardial work parameters. Other important variables, such as medication adherence, post-discharge interventions, and longitudinal functional recovery, were not assessed and may confound outcome interpretation.

Although BMI remains a widely used parameter for classifying obesity, it does not fully reflect the complexity of excess adiposity or its functional consequences. Recent expert recommendations [40] support a shift toward defining obesity based on adiposity-related organ dysfunction, incorporating parameters such as waist circumference, body fat percentage, or echocardiographic markers of fat distribution. Our study protocol was developed prior to the publication of these updated definitions and reflects the standard criteria in use at the time. Another relevant limitation is the lack of standardized protocols for myocardial work quantification in routine clinical practice. Although pressure–strain loop analysis using speckle-tracking echocardiography is increasingly utilized, variability in image acquisition, frame rate optimization, and post-processing software may hinder reproducibility across centers. To facilitate clinical translation, future studies should aim to standardize acquisition protocols and validate myocardial work indices in multicenter prospective cohorts.

## 5. Conclusions

LVGWE represents a novel and promising prognostic marker in patients with STEMI, particularly in those with obesity—a population known to exhibit more complex pathophysiological and clinical profiles. Its capacity to integrate myocardial deformation with energetic efficiency allows for a more comprehensive assessment of LV performance than traditional metrics. Incorporating LVGWE into routine post-STEMI evaluation may enhance risk stratification and enable more personalized management strategies, especially in high-risk subgroups.

## Figures and Tables

**Table 1:** Overview of clinical, biochemical, thrombolysis, and angiographic data.

Variable	All Patients (*n* = 143)	Non-Obese STEMI Patients (*n* = 90)	Obese STEMI Patients (*n* = 53)	*p* Value
Age, years	59 ± 11	60 ± 11	56 ± 10	0.035
Man, *n* (%)	111 (77.7)	71 (78.9)	40 (75.5)	0.636
Heart rate, bpm	82 ± 16	78 ± 15	83 ± 13	0.084
SBP, mmHg	146 ± 23	140 ± 23	155 ± 20	<0.001
DBP, mmHg	89 ± 15	86 ± 15	93 ± 14	0.011
Height, cm	170 (162–176)	170 (160–178)	171 (165–175)	0.678
Weight, kg	85 (71–104)	75 (65–87)	101 (96–114)	<0.001
BMI, kg/m^2^	28.80 (24.90–33.62)	25.40 (23.69–27.17)	34.25 (31.48–37.95)	<0.001
BSA, m^2^	2.06 (1.80–2.20)	1.85 (1.72–2.07)	2.20 (2.14–2.28)	<0.001
Creatinine level, mg/dL	1.08 (0.93–1.24	1.07 (0.84–1.27)	1.09 (1.00–1.18)	0.606
eGFR, ml/min/1.73 m^2^	75 ± 18	82.02 ± 21.11	83.13 ± 21.41	0.763
Glycemia, mg/dL	134 (111–168)	132 (109–154)	135 (113–217)	0.003
LDLc, mg/dL	117 ± 38	115.99 ± 30.76	121.57 ± 33.24	0.313
Triglycerides, mg/dL	129 (101–186)	103 (70–158)	154 (114–328)	0.006
Total cholesterol, mg/dL	185 ± 45	186.78 ± 37.74	190.68 ± 51.57	0.634
Hemoglobin, g/dL	14 ± 1.5	14.42 ± 1.51	14.96 ± 1.31	0.052
ESR, mm/h	12 (6–21)	12 (10–29)	10 (5–20)	0.443
Leukocytes, 10^3^ /µL	11.31 (9.54–13.72)	11.34 (8.88–12.55)	11.29 (9.81–14.78)	0.509
Neutrophiles, 10^3^ /µL	8.33 (6.68–10.54)	8.35 (7.03–9.82)	7.81 (6.58–12.04)	0.530
Peak troponin I, ng/L	1622 (220–23162)	5675 (287–34107)	445 (155–9715)	0.307
Peak CK-MB, U/L	222 (114–341)	222 (120–322)	212 (101–408)	0.524
Smoking, *n* (%)	98 (68.5)	64 (71.1)	34 (64.2)	0.387
Dyslipidemia, *n* (%)	137 (95.8)	85 (94.4)	52 (98.1)	0.291
Previous CAD, *n* (%)	9 (6.3)	5 (5.6)	4 (7.5)	0.726
Cardiac inheritance, *n* (%)	7 (4.9)	4 (4.4)	3 (5.7)	0.710
Diabetes, *n* (%)	30 (21)	9 (10.0)	21 (39.6)	<0.001
Hypertension, *n* (%)	108 (75.5)	59 (65.6)	49 (92.5)	<0.001
CKD, *n* (%)	16 (11.2)	9 (10.0)	7 (13.2)	0.557
Killip Class I, *n* (%)	120 (83.9)	75 (83.3)	45 (84.9)	0.805
Killip Class II, *n* (%)	20 (14)	12 (13.3)	8 (15.1)	0.769
Killip Class III, *n* (%)	2 (1.4)	2 (2.2)	0 (0.0)	0.530
Killip Class IV, *n* (%)	1 (0.7)	1 (1.1)	0 (0.0)	1.000
Thrombolysis, *n* (%)	39 (27.3)	26 (28.9)	13 (24.5)	0.572
Single-vessel disease, *n* (%)	63 (44.1)	36 (40.0)	27 (50.9)	0.203
Two-vessel disease, *n* (%)	47 (32.9)	31 (34.4)	16 (30.2)	0.601
Three-vessel disease, *n* (%)	33 (23.1)	23 (25.6)	10 (18.9)	0.359

Values are expressed as mean ± standard deviation or median (interquartile range). STEMI, ST-elevation myocardial infarction; SBP, systolic blood pressure; DBP, diastolic blood pressure; BMI, body mass index; BSA, body surface area; eGFR, estimated glomerular filtration rate; LDLc, low-density lipoprotein cholesterol; ESR, erythrocyte sedimentation rate; CK-MB, creatine kinase isoform MB; CAD, coronary artery disease; CKD, chronic kidney disease.

**Table 2:** Treatment at discharge.

Treatment at Discharge	All Patients (*n* = 143)	Non-Obese STEMI Patients (*n* = 90)	Obese STEMI Patients (*n* = 53)	*p* Value
Aspirin, *n* (%)	141 (98.6)	88 (97.8)	53 (100)	0.530
Prasugrel/ticagrelor, *n* (%)	109 (76.2)	68 (75.6)	41 (77.4)	0.807
Clopidogrel, *n* (%)	34 (23.8)	22 (24.4)	12 (22.6)	0.807
Betablocker, *n* (%)	109 (76.2)	62 (68.9)	47 (88.7)	0.007
Nitrate, *n* (%)	65 (45.5)	39 (43.3)	26 (49.1)	0.507
ACE inhibitor/ARBs *n* (%)	91 (63.6)	48 (53.3)	43 (81.1)	<0.001
MRAs, *n* (%)	114 (79.7)	71 (78.9)	43 (81.1)	0.747
Loop diuretics, *n* (%)	111 (77.6)	67 (74.4)	44 (83.0)	0.235
SGLT2i, *n* (%)	23 (16.1)	12 (13.3)	11 (20.8)	0.243
Statin, *n* (%)	143 (100)	90 (100)	53 (100)	

Values are expressed as percentages. STEMI, ST-elevation myocardial infarction; ACE, angiotensin-converting enzyme; ARBs, angiotensin II receptor blockers; MRAs, mineralocorticoid receptor antagonists; SGLT2i, sodium-glucose co-transporter 2 inhibitor.

**Table 3:** Transthoracic echocardiographic measurements: conventional 2D, tissue Doppler, and speckle tracking imaging.

Echocardiographic Parameter	All Patients (*n* = 143)	Non-Obese STEMI Patients (*n* = 90)	Obese STEMI Patients (*n* = 53)	*p* Value
Left ventricular end-diastolic volume, mL	107.76 ± 26.59	104.44 ± 29.12	117.00 ± 22.18	0.008
Left ventricular end-systolic volume, mL	63.47 ± 20.05	57.00 (44.50–72.50)	68.50 (62.00–75.00)	0.004
End-diastolic interventricular septal thickness, mm	1.19 ± 0.20	1.15 ± 0.17	1.27 ± 0.22	<0.001
End-diastolic posterior wall thickness, mm	1.12 ± 0.15	1.10 ± 0.15	1.21 ± 0.16	<0.001
Right ventricle diameter, cm	2.62 ± 0.32	2.58 ± 0.41	2.64 ± 0.32	0.370
Estimated pulmonary artery systolic pressure, mmHg	22 (21–29)	22 (21–29)	24 (20–29)	0.961
Left atrial volume index, mL/m^2^	25.88 (21.11–30.99)	24.82 (21.11–31.10)	26.21 (20.68–30.64)	0.278
Peak early diastolic mitral flow velocity (E), m/s	0.66 ± 0.18	0.65 ± 0.18	0.65 ± 0.19	0.916
Late transmitral flow velocity (A), m/s	0.78 ± 0.17	0.72 ± 0.19	0.82 ± 0.16	0.001
Mean peak early diastolic mitral annulus velocity (e’), m/s	0.07 (0.04–0.08)	0.07 (0.05–0.08)	0.06 (0.04–0.07)	0.579
Mean peak systolic mitral annulus velocity (s’), m/s	0.07 ± 0.01	0.07 ± 0.01	0.07 ± 0.01	0.380
E/e’ ratio	10.40 (8.33–13.41)	10.00 (8.30–11.81)	11.11 (8.33–14.89)	0.206
Left ventricular ejection fraction, %	41 ± 6	43 ± 7	43 ± 6	0.929
Global longitudinal strain, %	−12.03 ± 3.81	−12.97 ± 3.60	−12.08 ± 3.21	0.141

Values are expressed as mean ± standard deviation or median (interquartile range). STEMI, ST-elevation myocardial infarction; E/e’, ratio of early diastolic mitral inflow velocity (E) to peak early diastolic mitral annulus velocity (e’).

**Table 4:** Myocardial work parameters.

Myocardial Work Indices	All Patients (*n* = 143)	Non-Obese STEMI Patients (*n* = 90)	Obese STEMI Patients (*n* = 53)	*p* Value
Left ventricular global work index, mmHg%	1105 ± 456	1255 ± 440	1170 ± 420	0.263
Left ventricular global constructive work, mmHg%	1289 ± 445	1407 ± 427	1377 ± 407	0.680
Left ventricular global wasted work, mmHg%	149 (98–200)	133 (70–189)	155 (124–301)	0.005
Left ventricular global work efficiency, %	86 (78–91)	88 (80–93)	85 (75–87)	0.019

Values are expressed as mean ± standard deviation or median (interquartile range). STEMI, ST-elevation myocardial infarction.

**Table 5:** ROC analysis for the detection of MACEs in obese STEMI patients.

Parameters	AUC	95% CI	*p* Value
LVEF, %	0.540	0.329–0.751	0.709
GLS, %	0.610	0.405–0.814	0.293
Mean s’, m/s	0.664	0.466–0.861	0.104
E/e’	0.619	0.418–0.821	0.245
LA volume index, mL/m^2^	0.525	0.333–0.717	0.799
Estimated PASP, mmHg	0.606	0.426–0.785	0.249
LVGWI, mmHg%	0.621	0.381–0.860	0.323
LVGCW, mmHg%	0.589	0.367–0.810	0.431
LVGWW, mmHg%	0.688	0.503–0.872	0.046
LVGWE, %	0.736	0.559–0.914	0.009

ROC, receiver operating characteristics; MACEs, major adverse cardiac events; STEMI, ST-elevation myocardial infarction; AUC, area under the curve; CI, confidence interval; LVEF, left ventricular ejection fraction; GLS, global longitudinal strain; s’, peak systolic mitral annulus velocity; E/e’, ratio of early diastolic mitral inflow velocity (E) to peak early diastolic mitral annulus velocity (e’); LA, left atrium; PASP, pulmonary artery systolic pressure; LVGWI, left ventricular global work index; LVGCW, left ventricular global constructive work; LVGWW, left ventricular global wasted work; LVGWE, left ventricular global work efficiency.

**Table 6:** ROC analysis for the detection of MACEs in non-obese STEMI patients.

Parameters	AUC	95% CI	*p* Value
LVEF, %	0.493	0.348–0.638	0.921
GLS, %	0.522	0.380–0.638	0.758
Mean s’, m/s	0.474	0.340–0.608	0.104
E/e’	0.432	0.296–0.568	0.328
LA volume index, mL/m^2^	0.533	0.395–0.671	0.639
Estimated PASP, mmHg	0.521	0.385–0.657	0.762
LVGWI, mmHg%	0.568	0.423–0.712	0.359
LVGCW, mmHg%	0.556	0.416–0.697	0.433
LVGWW, mmHg%	0.581	0.435–0.728	0.277
LVGWE, %	0.578	0.440–0.716	0.269

ROC, receiver operating characteristics; MACEs, major adverse cardiac events; STEMI, ST-elevation myocardial infarction; AUC, area under the curve; CI, confidence interval; LVEF, left ventricular ejection fraction; GLS, global longitudinal strain; s’, peak systolic mitral annulus velocity; E/e’, ratio of early diastolic mitral inflow velocity (E) to peak early diastolic mitral annulus velocity (e’); LA, left atrium; PASP, pulmonary artery systolic pressure; LVGWI, left ventricular global work index; LVGCW, left ventricular global constructive work; LVGWW, left ventricular global wasted work; LVGWE, left ventricular global work efficiency.

**Table 7:** Univariate Cox regression analysis in obese STEMI patients.

Univariate Cox Regression	Hazard Ratio	95% CI	*p* Value
Age, years	1.026	0.964–1.092	0.418
BMI, kg/mp	0.966	0.820–1.139	0.680
Peak CK-MB, U/L	1.002	1.000–1.004	0.029
Peak troponin I, ng/L	1.000	1.000–1.002	0.144
LVEF, %	0.970	0.860–1.094	0.621
GLS, %	1.106	0.886–1.382	0.372
LVGWI, mmHg%	0.999	0.997–1.001	0.378
LVGCW, mmHg%	1.000	0.998–1.001	0.688
LVGWW, mmHg%	1.003	0.998–1.008	0.230
LVGWE, %	0.907	0.829–0.993	0.034
LVGWE < 79%	5.592	1.330–23.509	0.019
Multivessel coronary artery disease	2.669	0.638–11.176	0.179

BMI, body mass index; CK-MB, creatine kinase isoform MB; LVEF, left ventricular ejection fraction; GLS, global longitudinal strain; LVGWI, left ventricular global work index; LVGCW, left ventricular global constructive work; LVGWW, left ventricular global wasted work; LVGWE, left ventricular global work efficiency; CI, confidence interval.

**Table 8:** Univariate and multivariate Cox regression analysis for the entire study cohort.

Univariate Cox Regression				Multivariate Cox Regression		
	Hazard Ratio	95% CI	*p* Value	Hazard Ratio	95% CI	*p* Value
Age, years	1.017	0.985–1.050	0.312			
BMI, kg/mp	0.947	0.877–1.024	0.171			
Obesity status	0.621	0.276–1.395	0.248	0.534	0.233–1.223	0.138
Peak CK-MB, U/L	1.001	1.000–1.003	0.031	1.001	1.000–1.002	0.113
Peak Troponin I, ng/L	1.000	1.000–1.001	0.680			
LVEF, %	0.991	0.941–1.044	0.742			
GLS, %	1.038	0.935–1.153	0.485			
LVGWI, mmHg%	1.000	0.999–1.000	0.318			
LVGCW, mmHg%	1.000	0.999–1.001	0.529			
LVGWW, mmHg%	1.003	1.000–1.006	0.037			
LVGWE, %	0.957	0.921–0.995	0.025			
LVGWE < 79%	3.093	1.481–6.458	0.003	2.737	1.268–5.905	0.010
Multivessel coronary artery disease	2.253	1.068–4.755	0.033			

BMI, body mass index; CK-MB, creatine kinase isoform MB; LVEF, left ventricular ejection fraction; GLS, global longitudinal strain; LVGWI, left ventricular global work index; LVGCW, left ventricular global constructive work; LVGWW, left ventricular global wasted work; LVGWE, left ventricular global work efficiency; CI, confidence interval.

## Data Availability

The data presented in this study is available upon request from the corresponding author for the protection of the participants’ privacy.

## Abbreviations

The following abbreviations are used in this manuscript:

AUC	Area under the curve
BMI	Body mass index
GLS	Global longitudinal strain
LV	Left ventricle
LVEF	Left ventricular ejection fraction
LVGCW	Left ventricular global constructive work
LVGWE	Left ventricular global work efficiency
LVGWI	Left ventricular global work index
LVGWW	Left ventricular global wasted work
LVMW	Left ventricular myocardial work
MACE	Major adverse cardiac events
PCI	Percutaneous coronary intervention
ROC	Receiver operating characteristics
STEMI	ST-elevation myocardial infarction
TTE	Transthoracic echocardiography

## References

[1] Fruh S.M. (2017). Obesity: Risk factors, complications, and strategies for sustainable long-term weight management. J. Am. Assoc. Nurse Pract..

[2] GBD 2021 Adult BMI Collaborators (2025). Global, regional, and national prevalence of adult overweight and obesity, 1990–2021, with forecasts to 2050: A forecasting study for the Global Burden of Disease Study 2021. Lancet.

[3] Kolwicz S.C., Purohit S., Tian R. (2013). Cardiac metabolism and its interactions with contraction, growth, and survival of cardiomyocytes. Circ. Res..

[4] Cole M.A., Murray A.J., Cochlin L.E., Heather L.C., McAleese S., Knight N.S., Sutton E., Jamil A.A., Parassol N., Clarke K. (2011). A high fat diet increases mitochondrial fatty acid oxidation and uncoupling to decrease efficiency in rat heart. Basic Res. Cardiol..

[5] Huang J., Li G.A., Wang J., Jiao Y.W., Qian Z.F., Fan L., Tang L.M. (2023). Evaluation of subclinical left ventricular systolic dysfunction in obese patients by global myocardial work. Diabetol. Metab. Syndr..

[6] Parto P., Lavie C.J. (2017). Obesity and CardiovascularDiseases. Curr. Probl. Cardiol..

[7] Amir O., Elbaz-Greener G., Carasso S., Claggett B., Barbarash O., Zaman A., Christersson C., Kiatchoosakun S., Anonuevo J., Opolski G. (2025). Association between body mass index and clinical outcomes in patients with acute myocardial infarction and reduced systolic function: Analysis of PARADISE-MI trial data. Eur. J. Heart Fail..

[8] Basha M., Stavropoulou E., Nikolaidou A., Dividis G., Peteinidou E., Tsioufis P., Kamperidis N., Dimitriadis K., Karamitsos T., Giannakoulas G. (2025). Diagnosing Heart Failure with Preserved Ejection Fraction in Obese Patients. J. Clin. Med..

[9] Galderisi M., Cosyns B., Edvardsen T., Cardim N., Delgado V., Di Salvo G., Donal E., Sade L.E., Ernande L., Garbi M. (2017). 2016–2018 EACVI Scientific Documents Committee. Standardization of adult transthoracic echocardiography reporting in agreement with recent chamber quantification, diastolic function, and heart valve disease recommendations: An expert consensus document of the European Association of Cardiovascular Imaging. Eur. Heart J. Cardiovasc. Imaging.

[10] Russell K., Eriksen M., Aaberge L., Wilhelmsen N., Skulstad H., Remme E.W., Haugaa K.H., Opdahl A., Fjeld J.G., Gjesdal O. (2012). A novel clinical method for quantification of regional left ventricular pressure-strain loop area: A non-invasive index of myocardial work. Eur. Heart J..

[11] Edwards N.F.A., Scalia G.M., Shiino K., Sabapathy S., Anderson B., Chamberlain R., Khandheria B.K., Chan J. (2019). Global myocardial work is superior to global longitudinal strain to predict significant coronary artery disease in patients with normal left ventricular function and wall motion. J. Am. Soc. Echocardiogr..

[12] Ilardi F., D’Andrea A., D’Ascenzi F., Bandera F., Benfari G., Esposito R., Malagoli A., Mandoli G.E., Santoro C., Russo V. (2021). Myocardial Work by Echocardiography: Principles and Applications in Clinical Practice. J. Clin. Med..

[13] Papadopoulos K., Ozden T.O., Mitrousi K., Ikonimidis I. (2021). Myocardial work: Methodology and clinical applications. Diagnostics.

[14] El Mahdiui M., van der Bijl P., Abou R., Ajmone M.N., Delgado V., Bax J.J. (2019). Global Left Ventricular Myocardial Work Efficiency in Healthy Individuals and Patients with Cardiovascular Disease. J. Am. Soc. Echocardiogr..

[15] Marzlin N., Hays A.G., Peters M., Kaminski A., Roemer S., O’Leary P., Kroboth S., Harland D.R., Khandheria B.K., Tajik A.J. (2023). Myocardial Work in Echocardiography. Circ. Cardiovasc. Imaging.

[16] Zhao H., Jiang M., Wang W., Tao Z., Wang X., Chai Y., Han Y., Liu Q., Chen Y., Yue J. (2025). Subclinical myocardial work impairment in non-diabetic overweight and obese individuals: Impact of cardiometabolic traits. Int. J. Cardiol..

[17] Byrne R.A., Rossello X., Coughlan J.J., Barbato E., Berry C., Chieffo A., Claeys M.J., Dan G.A., Dweck M.R., Galbraith M. (2023). 2023 ESC Guidelines for the management of acute coronary syndromes. Eur. Heart J..

[18] Lang R.M., Badano L.P., Mor-Avi V., Afilalo J., Armstrong A., Ernande L., Flachskampf F.A., Foster E., Goldstein S.A., Kuznetsova T. (2015). Recommendations for cardiac chamber quantification by echocardiography in adults: An update from the American Society of Echocardiography and the European Association of Cardiovascular Imaging. Eur. Heart J. Cardiovasc. Imaging.

[19] Pasquali R., Casanueva F., Haluzik M., van Hulsteijn L., Ledoux S., Monteiro M.P., Salvador J., Santini F., Toplak H., Dekkers O.M. (2020). European Society of Endocrinology Clinical Practice Guideline: Endocrine work-up in obesity. Eur. J. Endocrinol..

[20] Lei Z., Li B., Li B., Peng W. (2022). Predictors and prognostic impact of left ventricular ejection fraction trajectories in patients with ST-segment elevation myocardial infarction. Aging Clin. Exp. Res..

[21] Yahud E., Tzuman O., Fink N., Goldenberg I., Goldkorn R., Peled Y., Lev E., Asher E. (2020). Trends in long-term prognosis according to left ventricular ejection fraction after acute coronary syndrome. J. Cardiol..

[22] Otero-García O., Cid-Álvarez A.B., Juskova M., Álvarez-Álvarez B., Tasende-Rey P., Gude-Sampedro F., García-Acuña J.M., Agra-Bermejo R., López-Otero D., Sanmartín-Pena J.C. (2021). Prognostic impact of left ventricular ejection fraction recovery in patients with ST-segment elevation myocardial infarction undergoing primary percutaneous coronary intervention: Analysis of an 11-year all-comers registry. Eur. Heart J. Acute Cardiovasc. Care.

[23] Holzknecht M., Reindl M., Tiller C., Reinstadler S.J., Lechner I., Pamminger M., Schwaiger J.P., Klug G., Bauer A., Metzler B. (2021). Global longitudinal strain improves risk assessment after ST-segment elevation myocardial infarction: A comparative prognostic evaluation of left ventricular functional parameters. Clin. Res. Cardiol..

[24] Akkuş Ö.F., Gürdoğan M. (2025). Effect of Global Longitudinal Strain at Discharge Period on Predicting Cardiac Defibrillator Implantation in STEMİ Patients with Impaired Left Ventricle Systolic Functions. Medicina.

[25] Lustosa R.P., Fortuni F., van der Bijl P., Mahdiui M.E., Montero-Cabezas J.M., Kostyukevich M.V., Knuuti J., Marsan N.A., Delgado V., Bax J.J. (2021). Changes in Global Left Ventricular Myocardial Work Indices and Stunning Detection 3 Months After ST-Segment Elevation Myocardial Infarction. Am. J. Cardiol..

[26] Lustosa R.P., van der Bijl P., El Mahdiui M., Montero-Cabezas J.M., Kostyukevich M.V., Ajmone Marsan N., Bax J.J., Delgado V. (2020). Noninvasive Myocardial Work Indices 3 Months after ST-Segment Elevation Myocardial Infarction: Prevalence and Characteristics of Patients with Postinfarction Cardiac Remodeling. J. Am. Soc. Echocardiogr..

[27] Sabatino J., De Rosa S., Leo I., Strangio A., Spaccarotella C., Polimeni A., Sorrentino S., Di Salvo G., Indolfi C. (2021). Prediction of Significant Coronary Artery Disease Through Advanced Echocardiography: Role of Non-invasive Myocardial Work. Front. Cardiovasc. Med..

[28] Pan J.C., Lyu L.J., Liu Q.D., Yang W., Li X.H., Han Y.M., Sun J.Y., Dong M., Zhang P.F., Zhang M. (2023). Association between resting myocardial work indices and stress myocardial perfusion in patients with angina and non-obstructive coronary artery disease. Quant. Imaging Med. Surg..

[29] Qin Y., Wu X., Wang J., Li Y., Ding X., Guo D., Jiang Z., Zhu W., Cai Q., Lu X. (2021). Value of territorial work efficiency estimation in non-ST-segment-elevation acute coronary syndrome: A study with non-invasive left ventricular pressure-strain loops. Int. J. Cardiovasc. Imaging.

[30] Lustosa R.P., Butcher S.C., van der Bijl P., El Mahdiui M., Montero-Cabezas J.M., Kostyukevich M.V., Rocha De Lorenzo A., Knuuti J., Ajmone M.N., Bax J.J. (2021). Global Left Ventricular Myocardial Work Efficiency and Long-Term Prognosis in Patients After ST-Segment-Elevation Myocardial Infarction. Circ. Cardiovasc. Imaging.

[31] Coisne A., Fourdinier V., Lemesle G., Delsart P., Aghezzaf S., Lamblin N., Schurtz G., Verdier B., Ninni S., Delobelle A. (2022). Clinical significance of myocardial work parameters after acute myocardial infarction. Eur. Heart J. Open.

[32] Ren J., Wu N.N., Wang S., Sowers J.R., Zhang Y. (2021). Obesity cardiomyopathy: Evidence, mechanisms, and therapeutic implications. Physiol. Rev..

[33] Koliaki C., Liatis S., Kokkinos A. (2019). Obesity and cardiovascular disease: Revisiting an old relationship. Metabolism.

[34] El Hadj Othmane T., El Hadj Othmane O., Nizar H. (2025). Obesity-Related Phenotype of Heart Failure with Preserved Ejection Fraction: A Comprehensive Review. Cureus.

[35] Taverna G., Trimarchi G., Lofrumento F., Mancinelli A., Teresi L., Alagna G., Carerj S., Zito C., Di Bella G. (2022). The impact of BMI on myocardial work and left atrial strain in young overweight patients with preserved left ventricular ejection fraction. Eur. Heart J..

[36] Alansari H., Lazzara G., Taha M.B., Gorthi J.R. (2025). The Impact of Obesity on Cardiovascular Diseases: Heart Failure. Methodist. Debakey Cardiovasc. J..

[37] Welsh A., Hammad M., Piña I.L., Kulinski J. (2024). Obesity and cardiovascular health. Eur. J. Prev. Cardiol..

[38] Abid A.R., El-Menyar A., Singh R., Gomaa M., Habib S., Abdelrahman A.S., Asaad N., AlQahtani A., Al-Thani H., AlBinali H. (2023). Patterns and Outcomes of Obesity Using Body Mass Index in Patients Hospitalized with Acute Cardiovascular Disorders: A Retrospective Analysis of 7284 Patients in a Middle Eastern Country. J. Clin. Med..

[39] Pop A.D., Pop D., Buzoianu A. (2020). Particularities of arrhythmias and obesity in heart failure. Rom. J. Cardiol..

[40] Rubino F., Cummings D.E., Eckel R.H., Cohen R.V., Wilding J.P.H., Brown W.A., Stanford F.C., Batterham R.L., Farooqi I.S., Farpour-Lambert N.J. (2025). Definition and diagnostic criteria of clinical obesity. Lancet Diabetes Endocrinol..

